# Metal oxide modified ZnO nanomaterials for biosensor applications

**DOI:** 10.1186/s40580-018-0159-9

**Published:** 2018-10-03

**Authors:** Nirmalya Tripathy, Deok-Ho Kim

**Affiliations:** 1 Department of Bioengineering, University of Washington, Seattle, WA 98109 USA; 2 Center for Cardiovascular Biology, University of Washington, Seattle, WA 98109 USA; 3 Institute for Stem Cell and Regenerative Medicine, University of Washington, Seattle, WA 98109 USA

**Keywords:** Metal oxide, ZnO, Nanomaterials, Biosensors, Enzymatic and non-enzymatic

## Abstract

Advancing as a biosensing nanotechnology, nanohybrids present a new class of functional materials with high selectivity and sensitivity, enabling integration of nanoscale chemical/biological interactions with biomedical devices. The unique properties of ZnO combined with metal oxide nanostructures were recently demonstrated to be an efficient approach for sensor device fabrication with accurate, real-time and high-throughput biosensing, creating new avenues for diagnosis, disease management and therapeutics. This review article collates recent advances in the modified ZnO nanostructured metal oxide nanohybrids for efficient enzymatic and non-enzymatic biosensor applications. Furthermore, we also discussed future prospects for nanohybrid materials to yield high-performance biosensor devices.

## Introduction

Nanoengineered biosensors have altered the field of biomedical engineering by introduction of smaller sensing structures for highly selective and sensitive detection of biomolecules [1]. Nanomaterials of varied forms, including single or hybrids/combinatorial nanostructures, can be designed with distinctive features that are significantly different from conventional bulk materials. With smart engineering, nanomaterials functionalities and characteristics can be optimized to allow high selectivity binding properties for analyzing nanoscale elements of biomolecules. Additionally, the distinct molecular recognition interactions at the nano-realm level in combination with nanoscale components can further promote high sensitivity of the biosensors [1]. Nanostructured metal oxides have received great attention for biosensing applications owing to several characteristics such as ease of fabrication and controllable size/shape, biocompatibility, catalytic and optical properties, chemical stability, strong adsorption ability and electron-transfer kinetics [2]. Zinc oxide (ZnO), an n-type semiconductor metal oxide with a wide direct band gap of 3.37 eV, a large exciton binding energy of 60 meV, and enhanced electron mobility, that has gathered attention for a broad range of applications in biomedical and clinical sciences. In addition, owing to its excellent film forming and adhesion capability, large surface area, strong adsorption ability due to high isoelectric point (~ 9.5), improved catalytic efficiency (oxygen storage capacity), better chemical stability, resistant against corrosion and oxidation, and small grain size, makes it highly amenable to biomolecular sensing applications [3–8]. Studies focusing on the morphological aspects of the nanostructured-ZnO have highlighted these nanostructures as advantageous because of high crystallinity with negligible structural defects and low-temperature synthesis. It also possesses good electrical conductivity which further makes it highly suitable for developing rapid, stable and reliable sensor devices [9, 10]. Furthermore, the biocompatible nature of ZnO nanostructures makes it a suitable choice for surface functionalization and interfacing with chemical/biological compounds at various temperature and pH levels [11].

Nanotextured surfaces coupled with metal oxides leads to the creation of nano-hybrid materials, which are expected to establish new avenues for both diagnosis and therapeutics because of these enhanced optical and electronic properties [12, 13]. Furthermore, computational modeling and experimental studies have also suggested that doping densities can greatly affect the sensitivity of bio-recognition [14, 15]. Hence, nanohybrids can be regarded as a promising new multifunctional material for high performance device fabrication. Modification of ZnO with metal oxide nanomaterials can further improve the features of ZnO for the sensing of biomolecules as metal oxide nanomaterials make great catalysts due to their high surface ratio of atoms with free valences of the total atoms in the cluster, which also may lead to electrochemical reversibility for redox reactions [16–18]. Therefore, the integration of ZnO with metal oxide nanomaterials can provide new avenues for the development of highly sensitive biosensors, where the surface functionalized-metal oxides serve as active sites for improving specificity and sensitivity, and the ZnO offers rapid electron transfer in an electrochemical reaction [19]. With regard to the material aspect of design choice, various metal oxide nanomaterials have been utilized for biosensing applications such as iron oxide, copper oxide, cerium oxide, magnesium oxide, and titanium oxide [20–25]. Owing to their size-dependent catalytic and optoelectronic properties, they can be tuned via size variations in nanoparticles, nanowires, nanotubes, nanorods, nanospheres, nanosheets, and quantum dots engineered through low temperature aqueous route, hydrothermal or solvothermal processes, sol–gel synthesis, or chemical vapor deposition are among available options [20–25].

Very recently, nanohybrids comprised of ZnO nanostructures coupled with metal oxide nanomaterials have attracted tremendous interest because of their potency for improving catalytic activity, surface-to-volume ratios and various other functionalities in a manner superior to pure ZnO nanomaterials. Thus, metal oxide modified ZnO nanostructure based biosensors have been highlighted to provide a new and efficient strategy for development of highly sensitive biosensors In this review article, we first discussed utilization of metal oxide modified ZnO nanomaterials for both enzymatic and non-enzymatic biosensor applications and summarized the advancement in sensing properties. Finally, we described the prospects for metal oxide modified ZnO nanomaterials in the context of further advancement in biosensing device fabrication.

## Metal oxide modified ZnO nanosheets based biosensor

### Electrochemical based non-enzymatic biosensor

**Fig. 1:**
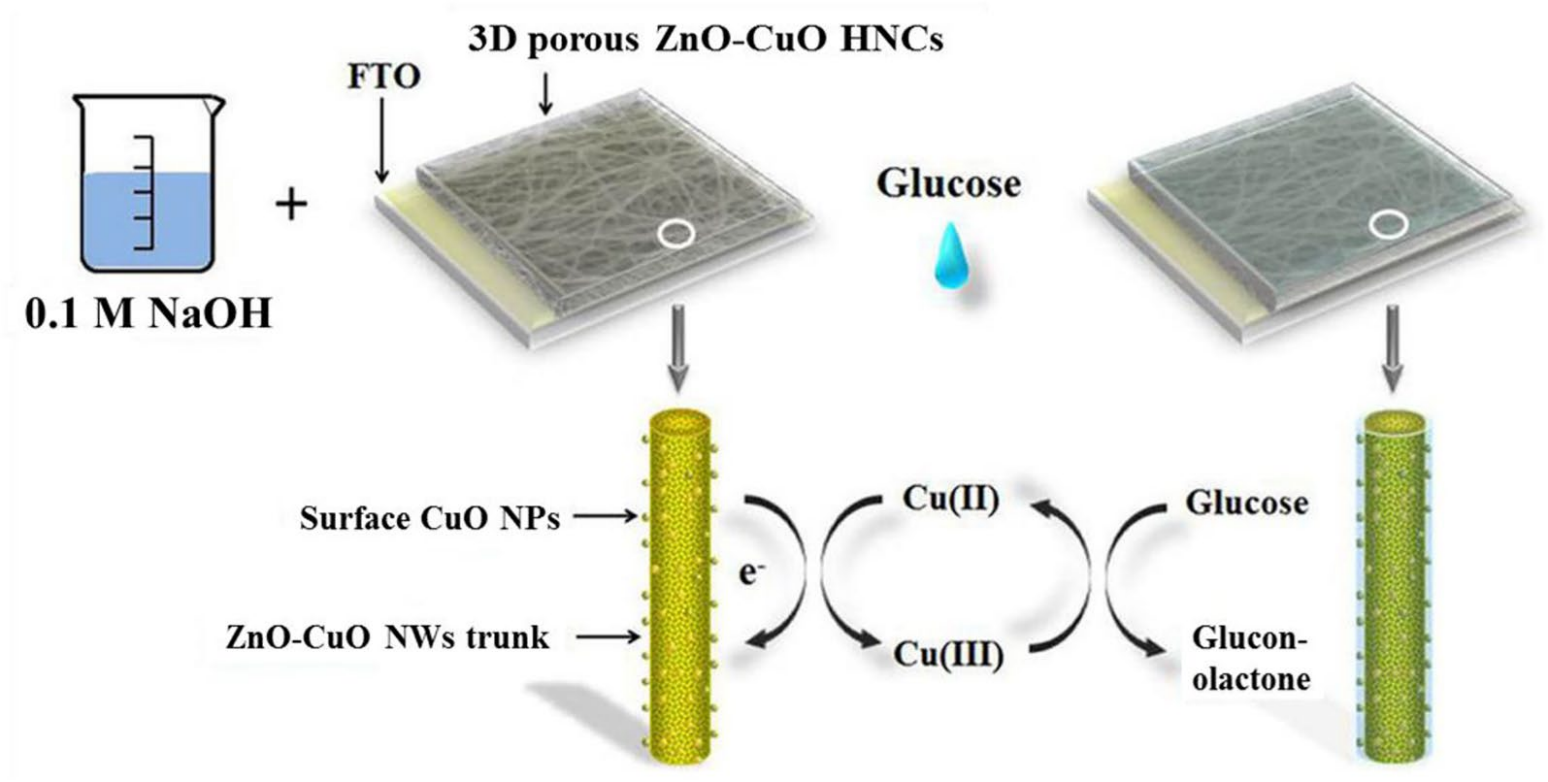
Schematic illustration of fabricated electrode and glucose detection mechanism over 3D porous ZnO–CuO HNCs surface (reproduced from Ref. [35] with permission, © 2014 Nature Publishing Group)

Although ZnO nanomaterials are very attractive for immobilization of low isoelectric enzymes during enzymatic based biosensor fabrications [26–33], ZnO nanomaterials are not considered to be a good candidate for non-enzymatic based biosensor fabrication due to its poor catalytic property towards biomolecules. Hence, it becomes necessary to modify ZnO nanostructure surfaces with a nanostructured transition metal oxide catalyst (such as CuO, Fe_2_O_3_, TiO_2_, Co_3_O_4_, NiO, NiWO_4_, etc.) or to make a nanocomposite of ZnO and metal oxide to catalyze the target analyte. Wu and Yin utilized an electrospinning method to deposit a nanostructured composite on platinum (Pt) electrodes [34]. Prior to sensing characterizations, the CuO–ZnO nanocomposites nanofibres were calcinated. Good sensing performance (i.e. low detection limit, high sensitivity and stability) for the non-enzymatic detection of glucose was obtained due to the synergistic effect between CuO nanofibers and ZnO. In another study, Zhou et al. also utilized an electrospinning method to deposit ZnO–CuO hierarchical nanocomposites on fluorine doped tin oxide (FTO) having porous and three-dimensional (3D) morphology for non-enzymatic biosensor fabrication [35]. The non-enzymatic biosensing properties of ZnO–CuO hierarchical nanocomposite/FTO revealed a significantly improved sensitivity of 3066.4 μA mM^−1^cm^−2^ and 0.21 μM detection limit (Fig. 1). Although the linear detection range (up to 1.6 mM) was low the electrospinning method produced reproducible and stable electrodes. The excellent sensing performance was attributed to the 3D porous morphology that offered a high surface area.

Vertically-grown/arrays-type orientations of nanostructures also have received attention due to their larger surface area. Soejima et al. synthesized CuO–ZnO composite nanoarrays using a facile one-step and low-temperature route on brass (Cu–Zn alloy) plates [36]. The synthesized nanoarrays were ZnO nanorods (NRs) and CuO nanoflowers. This nanocomposite based non-enzymatic sensor electrodes were electrocatalytically active during glucose oxidation and resulted in a fast response, low limit of detection and high sensitivity. Furthermore, SoYoon et al., and co-authors synthesized CuO nanoleaf–ZnO NRs hierarchical architectures and attached them to the Cu substrate to fabricate a non-enzymatic glucose sensor [37]. Excellent electrocatalytic activity of the nanohybrid composite (CuO nanoleaf–ZnO NRs) was obtained during glucose oxidation in NaOH buffer solution. As discussed earlier, improved performance was due to synergistic effect of CuO nanoleaf and ZnO NRs, which offered high electroactive surface area. Additionally, low working potential, good selectivity, low detection limit, and stable responses were shown in the results. However, this sensor resulted in a low dynamic detection range. In another interesting report, Karuppiah et al. also used a ZnO–CuO heterostructure to fabricate a non-enzymatic glucose biosensor using glassy carbon electrodes (GCE), which demonstrated an enhanced sensitivity, linear detection range, and a better detection limit [38]. Importantly, long-term stability of up to 38 days was achieved, which is acceptable for an assay for the non-enzymatic detection of glucose.

In another attempt to make a porous composite material with enhanced surface area, Cai et al. used a simple controllable top-down method to synthesize ZnO–CuO porous core–shell spheres [39]. The morphological characterization showed a core (CuO) surrounded by a porous shell (ZnO) with a large surface area, and thus the catalytic property of CuO and the electron transfer property of ZnO resulted in improved sensing performance. In another report, Marie et al. grew ZnO NRs vertically on FTO electrodes and functionalized with Fe_2_O_3_ to investigate the improvement as a non-enzymatic glucose sensor [40]. During electrode fabrication, they used a simple solution route to grow ZnO NRs followed by a surface dip-coating method to modify its surface with Fe_2_O_3_. Further, Nafion was coated to improve the selectivity of the non-enzymatic sensor in the presence of interfering species. Similarly, Strano and Mirabella grew ZnO NRs on Cu wire using a chemical bath deposition method and further modified with Ni(OH)_2_ flakes by pulsed electrodeposition [41]. ZnO NRs were vertically grown on Cu wire, which resulted in better stability of the electrode during non-enzymatic detection of glucose. This kind of small size working electrode is important for electrochemical measurements in small amounts of buffer solution.

**Fig. 2:**
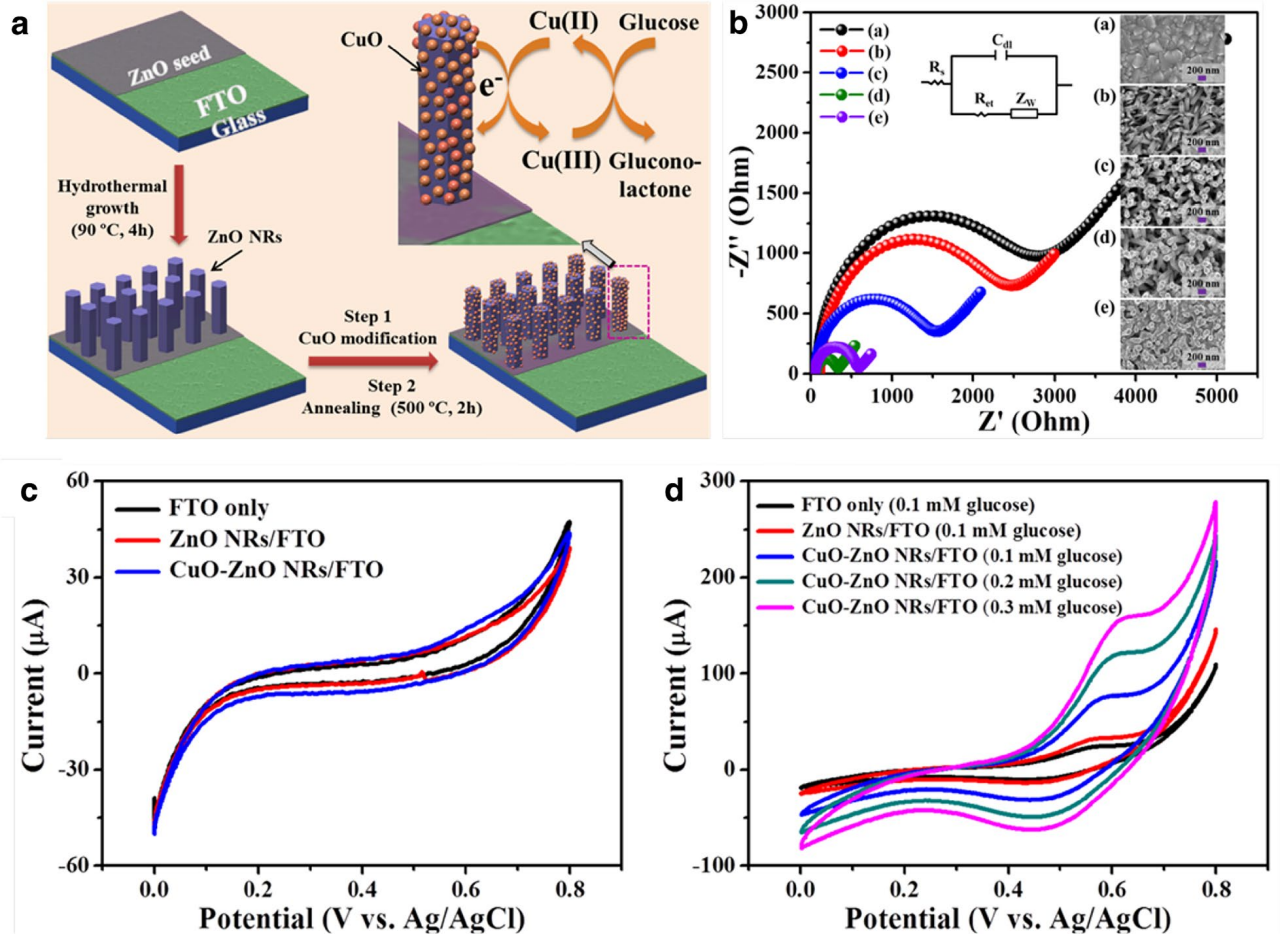
Schematic illustration and electrochemical characterizations. **a** Schematic of non-enzymatic glucose sensor fabrication. **b** Nyquist semicircle plots showing EIS spectra. **c** CV response of electrodes in blank NaOH solution at scan rate of 100 mVs^−1^. **d** CVs response of electrode in glucose solution (scan rate, 100 mVs^−1^) (reproduced from Ref. [42] with permission, © 2017 Nature Publishing Group)

**Fig. 3:**
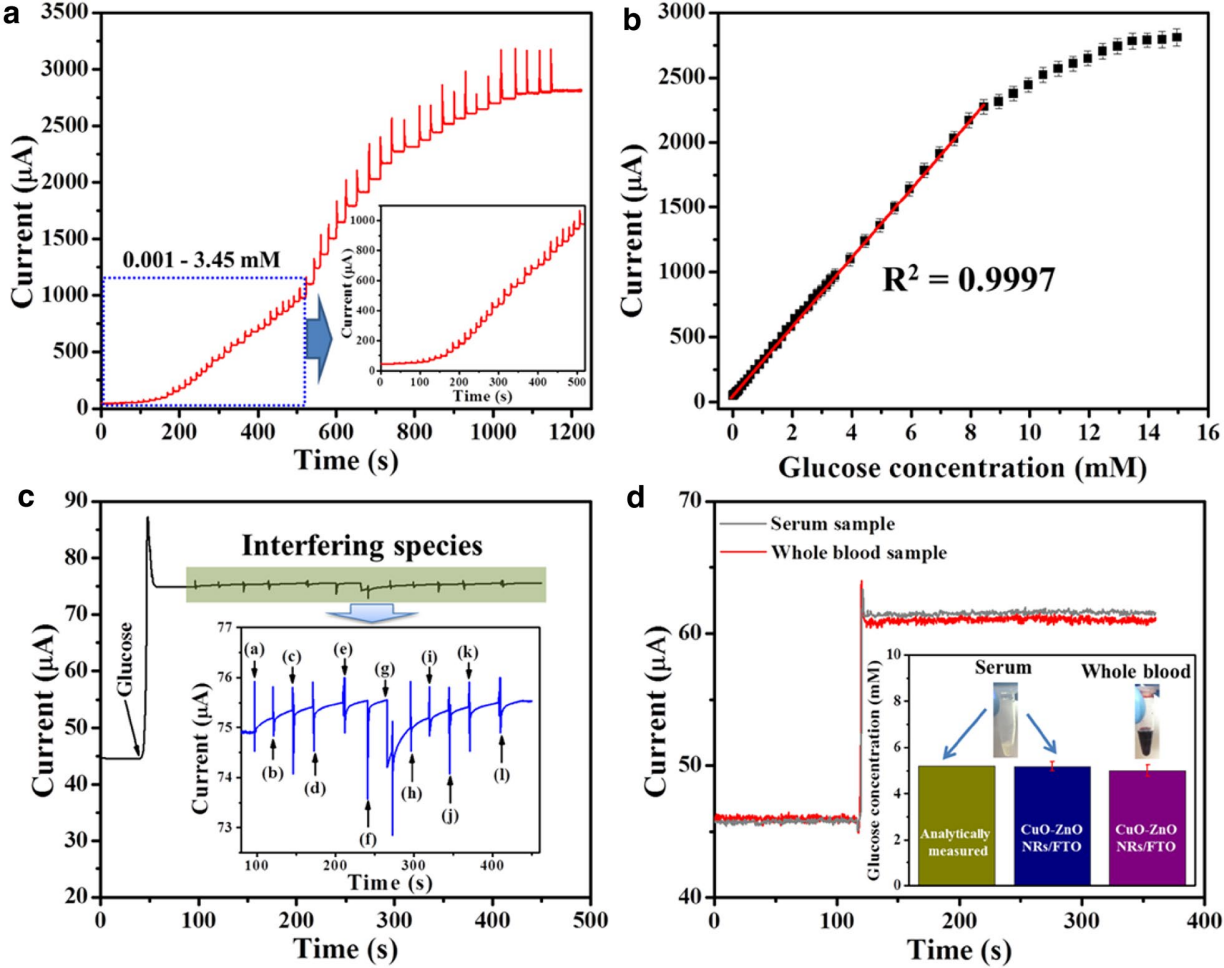
Electrochemical performance of biosensor. **a** Amperometric response of CuO–ZnO NRs/FTO electrode at different glucose concentration (0.001-4.95 mM). **b** Corresponding calibration plot. **c** Anti-interference ability test. **d** Real sample glucose detection (reproduced from Ref. [42] with permission, © 2017 Nature Publishing Group)

Recently, Ahmad et al. introduced a simple solution based method to fabricate a non-enzymatic glucose biosensor using vertically grown ZnO NRs on fluorine doped tin oxide (FTO) electrode after modifying the ZnO surface with CuO NPs [42]. Direct, vertically grown ZnO NRs using a low-temperature solution route offered excellent surface binding sites for CuO NPs loading. A schematic of the synthesis, modification, and detection mechanism is illustrated in Fig. 2a. Before biosensing characterization, the impedance spectra of the fabricated non-enzymatic glucose biosensor electrodes were measured to determine the optimal electrode for sensing performance (Fig. 2b). From the EIS spectra, it was clear that the 20 s CuO NPs modified ZnO NRs/FTO electrode showed a lower electron transfer resistance and further showing a diffusion-limited process between the electrode surface and the solution. The electrochemical tests of the optimized electrode were performed (Fig. 2c, d), using cyclic voltammetry (CV) in a three-electrode cell containing 0.1 M NaOH solution, working, reference, and counter electrodes. The blank samples showed no response, after subsequently adding glucose a clear oxidation peak at + 0.62 V was observed. When compared with bare ZnO NRs based electrodes, a clear oxidation peak obtained with CuO modified electrodes confirmed that the CuO NPs were the main catalyst, catalyzing the electro-oxidation of glucose. As reported previously [43], Cu(II) is electrochemically oxidized to Cu(III) during electrocatalytic oxidation of glucose and acts as an electron delivery system. Also, CV analysis at different scan rates suggested a linear increase in current response, which is a characteristic feature of surface-controlled electrochemical processes. A clear oxidation peak at + 0.62 V further demonstrated the analytical performance of the non-enzymatic glucose biosensor while examining different concentrations of glucose (Fig. 3a). The calibrated response plot (Fig. 3b) showed linearity up to a 8.45 mM glucose concentration, excellent sensitivity (2961.7 μA mM^−1^ cm^−2^), fast response time, and a low limit of detection (0.40 μM). Other important parameters (reproducibility, stability, reusability, and anti-interference ability) of the fabricated non-enzymatic biosensor were also evaluated. The excellent anti-interference ability of the biosensor in human serum samples, suggests its potential applicability in clinical samples (Fig. 3c, d). Collectively, the remarkable sensing performance of non-enzymatic biosensor was attributed to the directly-grown ZnO NRs holding CuO NPs that results in enhanced electrochemical activity for glucose and directly transfers electrons to the electrode surface. This fabrication method was simple and involves a completely low temperature process, which may provide a cost-effective fabrication method for glucose biosensors in biomedical applications.

**Fig. 4:**
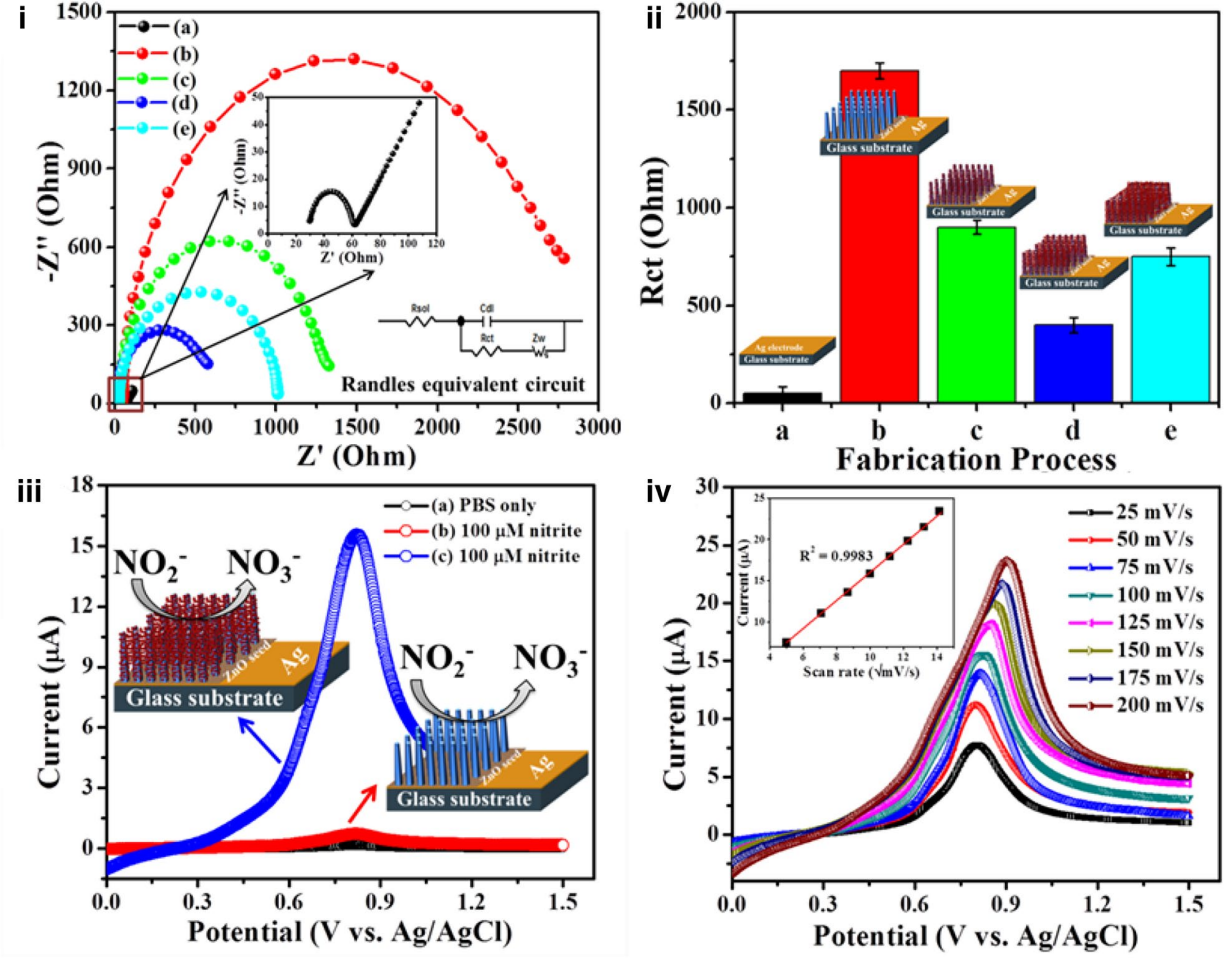
Electrochemical characterizations. **(i)** EIS analysis of *a* bare Ag, *b* ZnO NRs/Ag, *c* 1 min Fe_2_O_3_-ZnO NRs/Ag, *d* 2 min Fe_2_O_3_–ZnO NRs/Ag, and *e* 3 min Fe_2_O_3_-ZnO NRs/Ag in 5.0 mM [Fe(CN)6]^3−/4−^ solution with 100 mM KCl. **(ii)** Histogram showing Rct value of fabrication steps. **(iii)** LSVs without (*a*) and with 100 µM nitrite at ZnO NRs/Ag (*b*) and Fe_2_O_3_-ZnO NRs/Ag (*c*) in 0.1 M PBS. **(iv)** LSVs in 0.1 M PBS containing 100 µM nitrite at Fe_2_O_3_-ZnO NRs/Ag in the scan range of 25–200 mV/s. Insets of **(i)**, **(iii)**, and **(iv)** show the EIS spectrum of bare Ag electrode, nitrite detection schemes, and calibrated curve (reproduced from Ref. [44] with permission, © 2017 John Wiley & Sons)

**Fig. 5:**
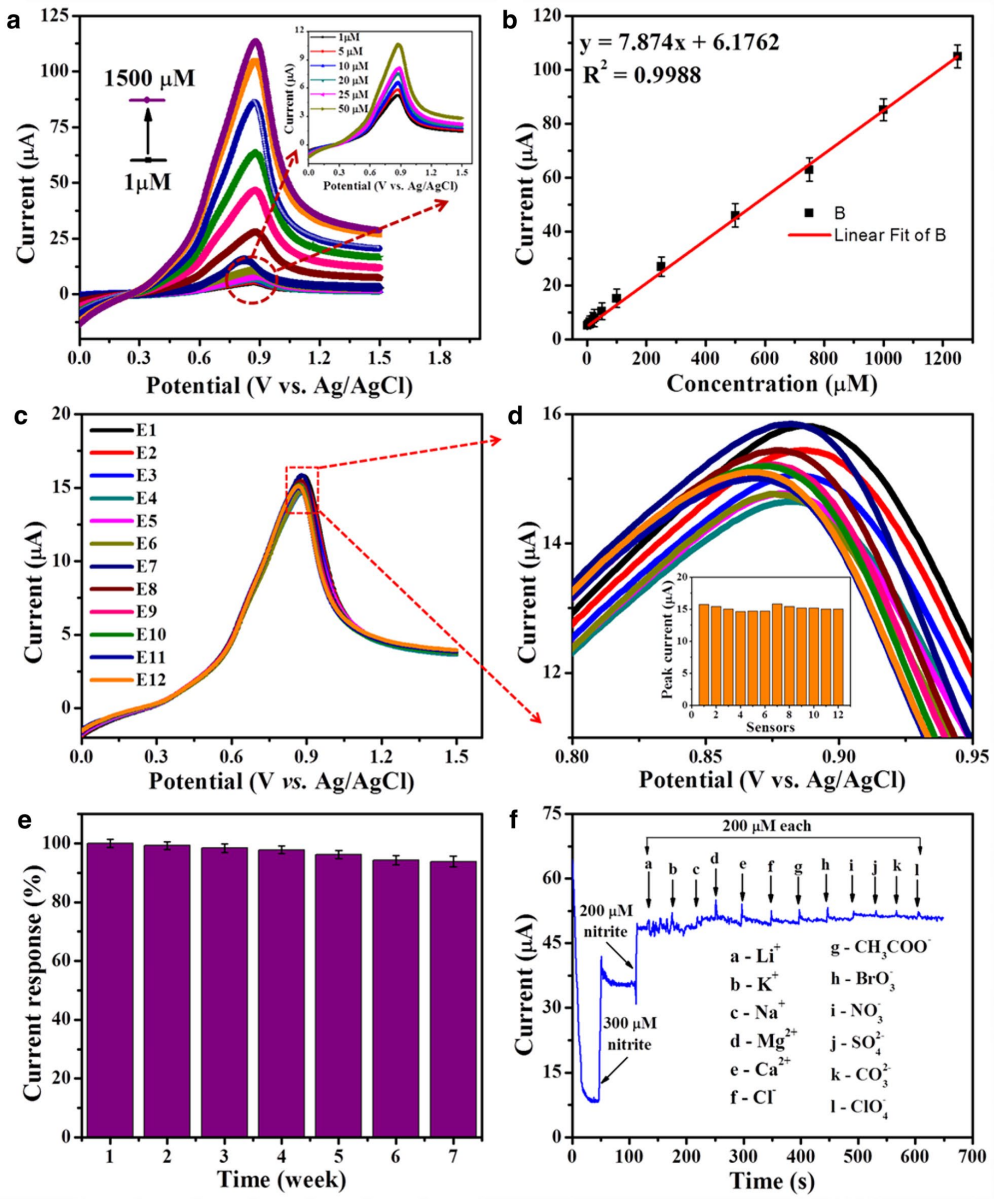
Biosensor device performance. **a** LSVs of Fe_2_O_3_ modified ZnO NRs/Ag electrode in different concentration (1–1500 µM) of nitrite. **b** Corresponding calibration curve. **c** LSVs of twelve optimized electrodes in PBS containing 100 µM nitrite. **d** Magnified peak curves. **e** Stability test. **f** Selectivity test (reproduced from Ref. [44] with permission, © 2017 John Wiley & Sons)

In another report, Ahmad et al. fabricated a non-enzymatic based nitrite sensor using Fe_2_O_3_ NPs coated ZnO NRs [44]. In this method, ZnO NRs were synthesized using a solution based approach and modified by dipping in a precursor solution followed by characterizations of the as-synthesized nanostructure material using X-ray diffraction (XRD) and field emission scanning electron microscopy (FESEM), which confirmed the synthesis and surface modification. To select the best performing sensor electrode, electrochemical impedance spectroscopy (EIS) was used to monitor the electrochemical behavior of the electrodes (Fig. 4i). Increasing surface modification with Fe_2_O_3_ NPs on ZnO NRs changes the impedance of fabricated electrodes and a lower charge transfer resistance was obtained for 2 min ZnO NR dipped in precursor solution due to excellent electron transfer between the redox probe and Ag electrode surface (Fig. 4ii). Linear sweep voltammograms (LSVs) were used to analyze the sensing characteristics of fabricated non-enzymatic sensors under optimized conditions. The LSV- showed that the bare ZnO NRs were not able to catalyze the nitrite; however a remarkably high current response was noticed after modification with Fe_2_O_3_ NPs (Fig. 4iii). The nitrite oxidation was a diffusion-controlled electrocatalytic process, which was confirmed after measuring LSV responses at different scan rates showing a linear increase in current *vs.* square root of scan rate (Fig. 4iv). The non-enzymatic nitrite sensor responded linearly with increasing nitrite concentration in buffer solution and showed high sensitivity (131.2 μAμM^−1^cm^−2^) in a wide linear range with a low detection limit of 15 nM (Fig. 5). These results can be attributed to the directly-grown ZnO NRs, surface modification with Fe_2_O_3_ NPs, and direct electron communication between the nanomaterials and electrode surface. Moreover, the ultralow limit of detection makes this non-enzymatic sensor a good choice for detecting low concentrations of nitrites in water and food samples.

### Non-enzymatic FET based biosensor

**Fig. 6:**
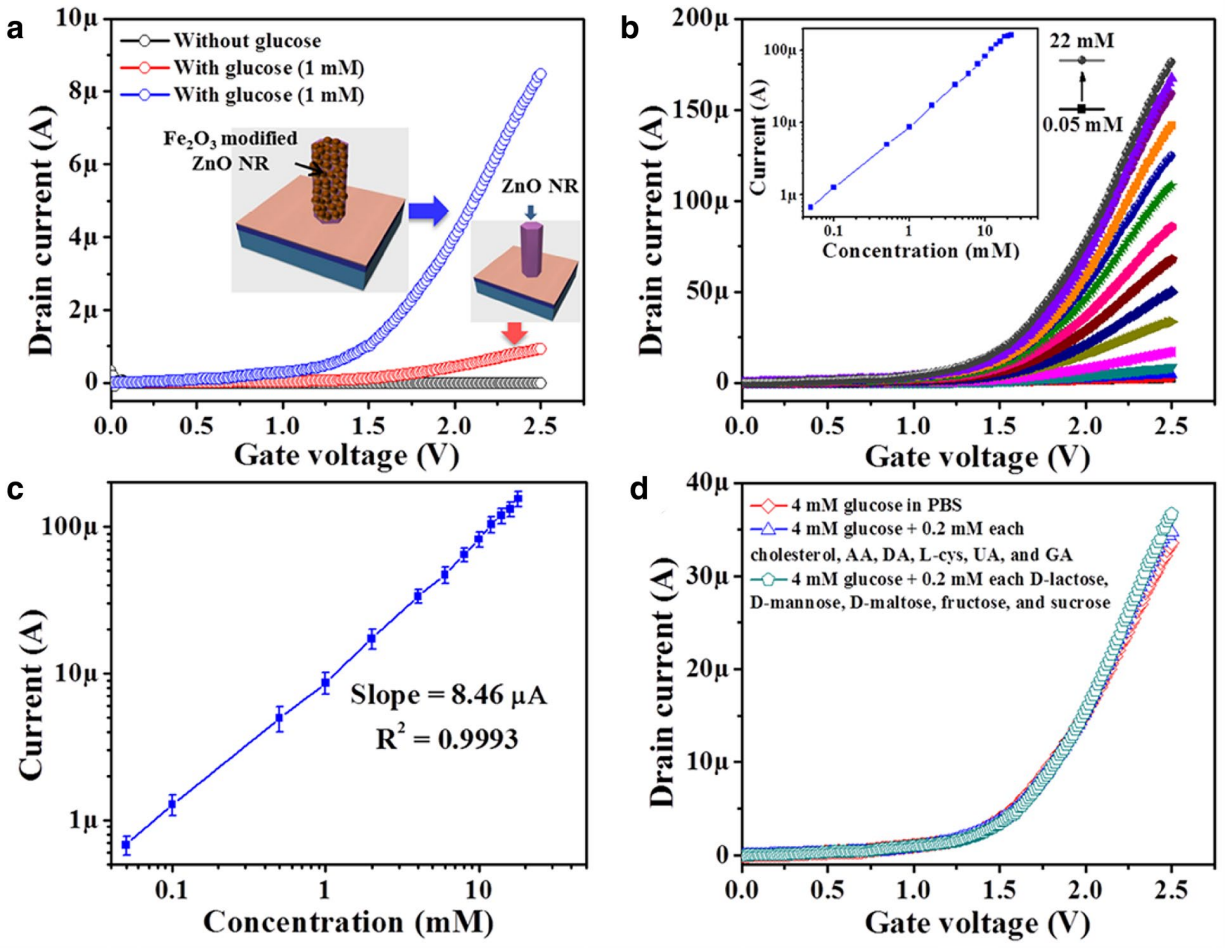
Sensing performance and selectivity test. **a** *I*–*V* response of FET-based non-enzymatic sensor in 0.1 mM PBS solution and 1 mM glucose contaning PBS. **b** *I*–*V* response in different concentrations of glucose. **c** Calibrated curve. **d** Selectivity test (reproduced from Ref. [47] with permission, © 2017 Elsevier)

Metal oxide modified ZnO nanomaterials were also used to fabricate field-effect transistor (FET) based biosensors. FET based biosensors are small, easy to operate, have a low fabrication cost, and need low operating potential. Recently, Jung and co-authors fabricated a flexible non-enzymatic FET based glucose biosensor after modifying ZnO NRs surfaces with NiO quantum dots (QDs) [45]. Although the sensitivity of this non-enzymatic FET sensor during glucose detection was low it showed a wide linear range, which is promising for detecting high glucose concentrations in serum samples without any sample dilution. Ahmad et al. fabricated another non-enzymatic FET based glucose biosensor using vertically oriented ZnO NRs [46]. As described previously, sensors based on only ZnO NRs were not able to catalyze glucose oxidation, resulting in a poor response current as compared to the Fe_2_O_3_ NPs modified ZnO NRs based FET device (Fig. 6a). The enhanced catalytic response was due to Fe_2_O_3_ NPs, which mediates the heterogeneous chemical oxidation/reduction of glucose. On the other hand, ZnO provided an excellent surface area for catalyst loading. The sensor showed linearity up to 18 mM, high and comparable sensitivity with CV and amperometric based detection methods (Fig. 6b, c). An excellent selectivity was also observed (Fig. 6d). Furthermore, 1 week stability and successful glucose detection in serum and whole blood make the sensor more useful for onsite glucose concentration detection.

### Enzymatic FET based biosensor

Recently, to detect specific species metal/metal oxide modified ZnO nanomaterials were further functionalized with enzymes/selective membranes. By doing this, an enhanced sensing performance was obtained. Ahn et al. immobilized valinomycin on Fe_2_O_3_ NPs-modified ZnO NRs to fabricate a FET based potassium sensor [47]. The analytical characterization of this sensor showed remarkably enhanced sensing response after Fe_2_O_3_ NPs modification as compared to the ZnO NRs based FET device. This result can be attributed to the high surface area of Fe_2_O_3_ NPs-modified ZnO NRs that favors increased valinomycin immobilization. ZnO NRs surface modification with Fe_2_O_3_ NPs also provides stability to the ZnO surface from acidic or basic solutions, which may be very useful for device stability during potassium detection. ZnO nanostructures are mostly grown on seeded substrates, however seed layers offer limited conductivity. The conductivity of electrode/seed layer holding nanostructures can also help to achieve improved sensitivity of the fabricated sensing device. Ahmad et al. designed a FET based calcium sensor device using a highly conductive seed layer [48]. First, they prepared a highly conductive seed layer on the substrate by subsequently depositing a ZnO seed layer, Ag nanowires, and then the ZnO seed layer again, which resulted in a sandwich-like layer. The first deposited seed layer was to provide stability by firmly attaching nanowires on the substrate and the second seed layer was used to grow ZnO NRs. During electrochemical characterizations, the role of every material was evaluated which confirmed that the introduction of Ag nanowires was the main contributing factor for enhancing device sensitivity. This study further offered an important strategy to enhance sensing performance which may be useful to design other biosensors with improved performance.

## Conclusion and future prospects

The rapid emergence of nanotechnology has led to the transition from single materials to nanohybrids including several interesting nanostructures such as nanorods, nanowires, hollow spheres, nanodisks, nanotubes, and nanobelts. These represent an expansion of potential detection candidates for fabrication of a wide range of biosensors, including electrochemical, enzymatic, and non-enzymatic biosensors. Key physiochemical and optical characteristics of nanohybrid materials include large surface area and high isoelectric point resulting in high adsorption efficiency, non-toxicity and biocompatibility, mechanical and chemical stability, catalytic efficiency (oxygen storage capacity), and reduction in potential, and electrical conductivity are advantageous for the development of an efficient and high-performance biosensor device. For example, fabrication of enzymatic biosensor devices with enhanced stability can be performed using directly-grown nanostructures on substrates followed by immobilization of specific biomolecules, while electrochemical biosensors can be developed via exploiting catalytic characteristics of various designed nanomaterials or nanostructures. The biosensors developed using ZnO-based nanostructures have immense potential for biomedical applications owing to their effective surface area combined with its biocompatible nature, ease of synthesis with controlled morphologies and pore sizes, and high electron communication. Further their high isoelectric point also ensures stable biomolecule immobilization while maintaining biological functionalities. Functionalization with metal oxides nanostructures not only improves the biosensor device stability, but also enhances selectivity, sensitivity and lowers detection limits of the desired biosensor. This article reviewed the current advancements in the development of metal-oxide modified ZnO nanostructure-supported biosensors with an emphasis on both enzymatic and non-enzymatic sensing devices for different analytes. Overall the appealing characteristics of metal oxides modified ZnO nanostructure based biosensors provide excellent advantages for designing sensors consisting of multifunctional and structural nanomaterials. Such nanohybrids based biosensors can be envisioned to revolutionize the fields of biomedical diagnostics, environmental remediation/monitoring, food safety testing among other applications.

The selection and design of nanomaterials is critical for rapid and accurate biomolecule detection. Thus, constant advancements in material synthesis approaches, enzyme/protein engineering and immobilization/conjugation strategies will continue to yield novel nano-engineered segments with improved functionality. Furthermore, an envisioned combination of newly emerging advanced manufacturing technologies which includes nanoscale-oriented three- or four-dimensional printing of multicomponent, multifunctional nanostructures are expected to bring new avenues to the present sensor design. Research in the field of advanced biosensing with biofunctionalized multifunctional nanomaterials, and the development of cost-effective biochip designs employing nanoscale sensing materials can further pave the way for nano-biosensing platforms and the realization of an economical lab-on-a-chip replacement for real- or near real-time biomolecules sensing.
